# The Nrf2 Activator CDDO-Imidazole Suppresses Inflammation-Induced Red Blood Cell Alloimmunization

**DOI:** 10.3390/antiox14060678

**Published:** 2025-06-03

**Authors:** Che-Yu Chang, Rosario Hernández-Armengol, Kausik Paul, June Young Lee, Karina Nance, Tomohiro Shibata, Peibin Yue, Christian Stehlik, David R. Gibb

**Affiliations:** 1 Department of Pathology and Laboratory Medicine, Cedars-Sinai Medical Center, Los Angeles, CA 90048, USA; che-yu.chang@cshs.org (C.-Y.C.); rosario.hernandezarmengol@cshs.org (R.H.-A.); kausik.paul@cshs.org (K.P.); jlee577951@student.wmcarey.edu (J.Y.L.); christian.stehlik@cshs.org (C.S.); 2 Department of Biomedical Sciences, Cedars-Sinai Medical Center, Los Angeles, CA 90048, USA; karina.nance@cshs.org; 3 Department of Pharmacology, Yokohama City University School of Medicine, Yokohama 236-0004, Japan; shibata.tom.wz@yokohama-cu.ac.jp; 4 Department of Medicine, Division of Hematology and Oncology, Cedars-Sinai Medical Center, Los Angeles, CA 90048, USA; peibin.yue@cshs.org; 5 Division of Transfusion Medicine, Cedars-Sinai Medical Center, Los Angeles, CA 90048, USA

**Keywords:** RBC alloimmunization, type 1 interferons, transfusion, Nrf2, inflammation

## Abstract

Experimental Objective: During red blood cell (RBC) transfusion, inflammation promotes the production of anti-RBC alloantibodies that can cause significant hemolytic events. Avoiding RBC antigen exposure is the only strategy to prevent RBC alloimmunization in transfusion recipients. Identifying mechanisms that inhibit alloimmunization may lead to novel prophylactic interventions. One potential regulatory mechanism is the activation of the transcription factor nuclear factor erythroid-derived 2-like 2 (Nrf2), a master regulator of antioxidant pathways. Pharmacologic Nrf2 activators induce antioxidant production and improve the sequelae of inflammatory diseases. Thus, we tested the hypothesis that a Nrf2 activator, 1-[2-cyano-3-,12-dioxooleana-1,9(11)-dien-28-oyl]-imidazole (CDDO-Im), regulates inflammation-induced RBC alloimmunization. Methods: WT and *Nrf2*-deficient mice were treated with inflammatory stimuli and CDDO-Im prior to transfusion with RBCs expressing the KEL antigen (KEL+ RBCs). Anti-KEL IgM and IgG were measured in the serum of transfused mice. Nrf2-activated gene expression and interferon activity were measured in mice and human macrophages pre-treated with CDDO-Im and interferon stimuli. Results: Here, we report that CDDO-Im induces Nrf2-activated gene expression and inhibits type 1 interferon activity, which promotes RBC alloimmunization in transfusion models. In mice transfused with KEL+ RBCs, pre-treatment with CDDO-Im inhibited inflammation-induced anti-KEL antibody production and increased the post-transfusion recovery of KEL+ RBCs in a Nrf2-dependent manner. CDDO-Im also inhibited RBC alloimmunization in mice with pre-existing inflammation. Conclusions: These results indicate that the activation of the Nrf2 antioxidant pathway regulates RBC alloimmunization to the KEL antigen in a pre-clinical model. If these findings translate to other models and human studies, Nrf2 activators may represent a potential prophylactic intervention to inhibit alloimmunization.

## 1. Introduction

During red blood cell (RBC) transfusion, most non-ABO antigens are not routinely matched between donors and recipients. This exposure of mis-matched antigens can cause the production of anti-RBC alloantibodies, which mediate hemolytic events including hemolytic transfusion reactions, which are a cause of transfusion-associated mortality [1,2]. Transfusion-dependent patients commonly produce alloantibodies against multiple RBC antigens. Acquiring compatible RBCs lacking many RBC antigens for these patients can be difficult if not unachievable. Thus, they commonly experience anemia-induced morbidities and/or receive incompatible RBC transfusions in the presence of anti-RBC antibodies, which can cause hemolytic transfusion reactions [3,4].

Avoiding RBC antigen exposure is the only clinically used strategy to prevent RBC alloimmunization in transfusion recipients. The provision of extended antigen-matched RBCs (i.e., the matching of C, E, and KEL antigens in addition to ABO/Rh(D)) has significantly reduced the frequency of alloimmunization in patients with hemoglobinopathies [5]. However, extended antigen matching is not utilized universally [6]. In addition, as there are as many as 340 antigens on the RBC surface and there are numerous variants of the Rh antigens (D, C, and E) [7,8], other strategies to inhibit alloimmunization are needed.

One factor that influences RBC alloimmunization is inflammation in the transfusion recipient. Patients with disseminated viral infections or inflammatory autoimmune diseases have elevated frequencies of alloimmunization [9,10,11,12,13]. In addition, many patients with sickle cell disease (SCD) have a high transfusion burden and inflammation due to leukocytosis, intravascular endothelial and myeloid cell activation, and vaso occlusion [14]. Patients with SCD have the highest frequency of RBC alloimmunization [10], and patients transfused during a vaso occlusive crisis or acute chest syndrome have profoundly increased odds of alloimmunization compared to patients with SCD in their baseline state of health [5]. We and others have used pre-clinical murine models to investigate mechanisms of inflammation-induced alloimmunization. Viral infection and treatment with the viral mimetic polyinosinic:polycytidylic acid (poly(I:C)) before transfusion enhance RBC alloimmunization in murine transfusion models [15,16,17,18]. We have reported that viral-induced type 1 interferons (IFNα/β) are required for alloimmunization in multiple murine transfusion models [17,18,19,20], and recombinant IFNα (rIFNα) is sufficient to induce alloimmunization [18].

Unlike patients with inflammation, the incidence of alloimmunization in the general transfused population is low. RBC alloimmunization only occurs in 3–10% of patients in US hospitals [10]. This extends to transfusion-dependent patients with higher frequencies of alloimmunization. While 30–50% of patients with SCD produce RBC alloantibodies, many recipients with SCD do not, despite having a high transfusion burden [10,14]. This indicates that there may be negative regulatory mechanisms that prevent alloimmunization. Such mechanisms could be leveraged for prophylactic interventions to prevent alloimmunization.

One potential regulatory mechanism is the activation of the transcription factor nuclear factor erythroid-derived 2-like 2 (Nrf2), which is a master regulator of antioxidant pathways activated by oxidative stress. While Nrf2 is recognized for its regulatory role in tumor cell proliferation and invasion [21], its role in transfusion is not known. At a steady state, Nrf2 is associated with its principal negative regulator, Kelch-like ECH-associated protein 1 (Keap1), in the cytoplasm. During oxidative stress, Nrf2 dissociates from Keap1, translocates to the nucleus, and induces the expression of antioxidant enzymes, including heme oxygenase 1 (HMOX1) and NAD(P)H quinone dehydrogenase 1 (NQO1). Nrf2 also regulates iron metabolism and inflammatory responses. During blood transfusion or heightened states of RBC phagocytosis by macrophages, the release of oxidized heme in the cytoplasm causes oxidized stress, leading to Nrf2 activation and HMOX1 activation, which catabolizes oxidized heme [22]. HMOX1 has anti-inflammatory functions and Nrf2 can directly inhibit inflammatory cytokine gene transcription [23,24]. Nrf2 has been shown to suppress inflammatory responses in murine models of inflammation, including sepsis [25], neuroinflammation [26], hepatic disease [27] and viral infection [28], and there is growing evidence that Nrf2 regulates IFNα/β responses. Gunderstofte et al. reported that Nrf2 down-regulation or deficiency in bone marrow-derived macrophages (BMDMs) results in elevated IFNα/β and the expression of IFNα/β-stimulated genes (ISGs) that are protective in a model of herpes simplex virus-2 [28]. A similar inverse correlation between Nrf2 activation and ISG expression was observed in human epithelial cells treated with Nrf2 siRNA [29].

Due to the antioxidant and anti-inflammatory roles of Nrf2, pharmacologic Nrf2 activators have been tested in many inflammatory conditions. Some have entered clinical trials and are FDA-approved for specific indications [30,31]. Most activators are electrophilic compounds that alter cysteines of Keap1, allowing the release and activation of Nrf2 [32]. CDDO-Im (1-[2-cyano-3-,12-dioxooleana-1,9(11)-dien-28-oyl]-imidazole) is a synthetic triterpenoid derived from oleanolic acid and a potent Nrf2 activator [33]. Nrf2 activators, including CDDO-Im, sulforaphane, and dimethyl fumarate (DMF), were shown to inhibit vaso occlusion and vascular inflammation in models of SCD [34,35,36]. However, the role of Nrf2 activators in regulating RBC alloimmunization has not been previously investigated. Here, we aimed to determine the role of CDDO-Im-induced Nrf2 activation in regulating IFNα/β responses in human macrophages and regulating RBC alloimmunization in a pre-clinical transfusion model.

Limitations of the study include the use of one Nrf2 activator and a transfusion model with one RBC alloantigen. Future studies will determine if the results extend to alternate activators and other RBC antigens. In addition, while Nrf2 activation occurs readily in macrophages [37], the effects of Nrf2 activation in other cell types on RBC alloimmunization will await studies in cell-specific Nrf2-deficient mice.

## 2. Materials and Methods

### 2.1. CDDO-Im Administration to Mice

C57BL/6J and *Nrf2*^-/-^ mice were purchased from Jackson Laboratories (Bar Harbor, ME, USA). K1 transgenic mice expressing the human KEL glycoprotein containing the KEL1 antigen on RBCs were previously described [18]. C57BL/6J and *Nrf2*^-/-^ mice, 8–12 weeks of age, were intraperitoneally injected with 2.5 to 10 mg/kg of CDDO-Im (Tocris Bioscience, Bristol, UK). All animal procedures (Protocol 8095) were approved by the Cedars-Sinai Institutional Animal Care and Use Committee on 31 May 2024.

### 2.2. RBC Transfusion

KEL+ and wildtype (WT) RBCs were collected from K1 and C57BL/6 mice, respectively, by phlebotomy and anticoagulated with 12% Citrate Phosphate Dextrose Adenine (CPDA-1, Jorgensen Labs, Melville, NY, USA). RBCs were leukoreduced with leukoreduction syringe filters (Pall, East Hills, NY, USA) and resuspended in PBS. In total, 50 µL of packed RBCs, the approximate murine equivalent of one unit of human RBCs, were transfused via retroorbital or tail vein injection. There were no adverse events from retroorbital transfusion. Recipient mice were pre-treated with or without CDDO-Im and/or 100 µg poly(I:C) (Invivogen, San Diego, CA, USA) by i.p. injection 3–6 h prior to transfusion.

### 2.3. Anti-KEL Antibody Measurement

Serum was collected from transfused mice 5, 7 and 14 days after transfusion. Anti-KEL IgM and anti-KEL IgG were measured by flow cytometric crossmatch, as previously described [20], 5 and 7–14 days after transfusion with KEL+ RBCs, respectively. Briefly, KEL+ RBCs from non-transfused mice were incubated with transfusion recipient serum, washed, and stained with secondary antibodies, goat anti-mouse IgG APC or IgM FITC (Jackson ImmunoResearch, West Grove, PA, USA). The mean fluorescence intensity (MFI) was measured on a Cytek Northern Lights flow cytometer (Fremont, CA, USA). The adjusted MFI was calculated by subtracting the reactivity of serum with WT RBCs from serum reactivity with KEL+ RBCs.

### 2.4. Post-Transfusion Recovery

Mice previously transfused with KEL+ RBCs were re-transfused retro-orbitally with a 1:1 mixture of KEL+ RBCs and WT RBCs 35 days after the initial transfusion. KEL+ RBCs were labeled with fluorescent 1,1′-dioctadecyl-3,3,3′,3′-tetramethylindocarbocyanine perchlorate (DiI) and WT RBCs were labeled with 3,3′-dioctadecyloxacarbocyanine perchlorate (DiO, Life Technologies, Camarill, CA, USA). DiI- and DiO-labeled RBCs remaining in circulation were quantified by flow cytometry 10 min and 1–3 days after transfusion. The ratio of KEL+: WT RBCs on days 1–3 was plotted as a percentage of the ratio measured 10 min after transfusion as previously described [20].

### 2.5. CDDO-Im Treatment of Human Macrophages

Leukoreduction System cones were obtained following apheresis platelet donation by de-identified platelet donors in the Cedars-Sinai Blood Donation Center. Peripheral blood mononuclear cells were isolated by a Ficoll-Paque Premium density gradient (Cytiva, Marlborough, MA, USA) and monocytes were enriched by magnetic negative selection using an EasySep human monocyte isolation kit (StemCell Technologies, Vancouver, CA, USA). Monocytes were differentiated into macrophages with GM-CSF (50 ng/mL) in serum-free macrophage media SFM (Thermo Fisher Scientific, Waltham, MA, USA) containing penicillin–streptomycin (P/S, 10 U/mL) for 5 days. Macrophages were then treated with CDDO-Im (0.2–0.8 µM) or sulforaphane (5–10 µM) for 18 h in complete RPMI media containing 10% FBS, 1% L-glutamine, 1% P/S, 1% non-essential amino acids, 1% Sodium Pyruvate, and 1% HEPES (Thermo Fisher Scientific). For some experiments, macrophages were then washed and cultured with 1 µg/mL poly(I:C) in complete RPMI for 3–24 h.

### 2.6. Flow Cytometry of Human Macrophages

Cultured human macrophages were collected using trypsin-EDTA (0.25%, Thermo Fisher, St. Bend, OR, USA). Fc receptors were blocked using human TruStain FcX and then labeled with mouse anti-human Siglec-1 PE, CD14 PerCP, CD64 BV785, CD38 APC and Zombie NIR (Biolegend, San Diego, CA, USA). Cells were then fixed and permeabilized using the Cyto-Fast Fix/Perm Buffer set (Biolegend) and stained with rat anti-mouse HMOX1 (MA1-112, Thermo Fisher Scientific), which was conjugated to Alexa Fluor 405 using the Zenon mouse IgG1 labeling kit according to manufacturer’s instructions (Thermo Fisher Scientific). Macrophages were analyzed with a Cytek Northern Lights flow cytometer.

### 2.7. RT-QPCR

RNA was isolated from human macrophages and murine blood leukocytes using a Qiagen RNeasy mini-kit (Hilden, DE, Germany) and reverse transcribed into cDNA with the Maxima H Minus cDNA Synthesis Master mix (Thermo Fisher Scientific). cDNA encoding mouse *HMOX1*, *NQO1*, and *GAPDH*, and human *AKR1C1*, *HMOX1*, *NQO1*, *MXA*, *CXCL10* (*IP-10*), *ISG15*, and *GAPDH* was measured using the PowerUp SYBR Green master mix on the QuantStudio 5 Real-Time PCR System (Thermo Fisher Scientific). Primer sequences are listed in Supplementary Table S1. The relative expression of target genes, compared to *GAPDH*, was determined using Thermo Fisher Connect software (applied biosystems™ analysis software, standard curve analysis module, version 3.9). Relative gene expression was calculated as Relative Expression = 2^−ΔCt^ × 1000, where ΔCt = target gene Ct − *GAPDH* Ct. For the expression of *MXA*, *IP-10*, and *ISG15* in human macrophages, relative expression is expressed as fold expression relative to the untreated group. Fold expression = the relative expression of the target gene in the treated group/the relative expression of the target gene in the untreated group.

### 2.8. Cytokine Quantification

Mouse serum cytokines were measured using the LEGENDplex mouse anti-virus response bead assay according to manufacturer’s instructions (Biolegend). Samples were analyzed on a Cytek flow cytometer and cytokine levels were calculated using the LEGENDplex Data Analysis Software Suite (version 2024-06-15).

### 2.9. Statistical Analysis

GraphPad Prism 10 was used for statistical analysis. For comparing anti-RBC antibody and post-transfusion recovery data, non-parametric testing with the Mann–Whitney U test (2 groups) or the Kruskal–Wallis with Dunn’s post-test (>2 groups) was performed. For RT-qPCR, flow cytometry, and cytokine data, parametric testing with Student’s *t*-test or a one-way ANOVA with Tukey’s post-test was used for comparing 2 or more than 2 samples, respectively. *p*-values < 0.05 were considered statistically significant. Post-transfusion recovery graphs show the mean as a symbol. Error bars represent standard deviation. Bar graphs show the mean as a vertical bar and individual mice or human samples as a white circle.

## 3. Results

### 3.1. CDDO-Im Induces Expression of Nrf2-Stimulated Genes in Murine Blood Leukocytes

**Figure 1:**
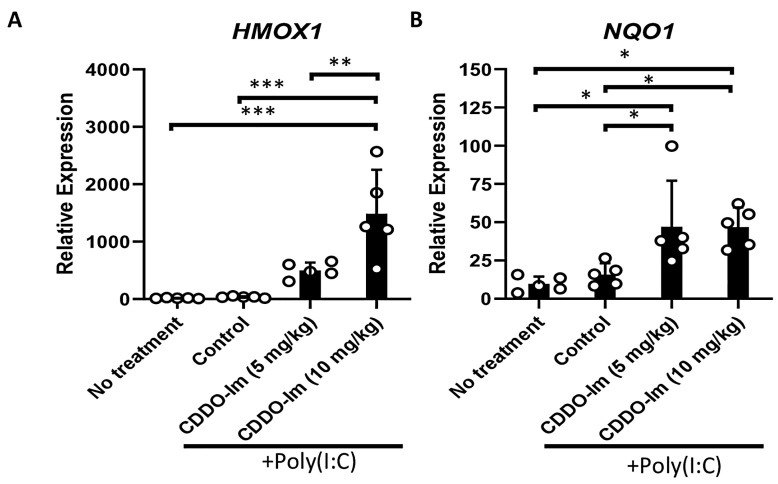
CDDO-Im induces Nrf2-activated gene expression in mice. WT mice were treated (i.p.) with 5–10 mg/kg CDDO-Im and/or 100 µg poly(I:C) 6 h and 3 h prior to analysis, respectively. (**A**) *HMOX1* and (**B**) *NQO1* expression by blood leukocytes measured by RT-qPCR. * *p* < 0.05, ** *p* < 0.01, *** *p* < 0.001 by one-way ANOVA with Tukey’s post-test; 5 mice per group; representative of 3 independent experiments.

CDDO-Im has been shown to be a potent activator of Nrf2 signaling [33]. Thus, we initially verified that the CDDO-Im treatment of wildtype (WT) mice, 6 h prior to analysis, induces the expression of Nrf2-stimulated antioxidant genes, *HMOX1* and *NQO1*, in blood leukocytes. Given that poly(I:C) was utilized in transfusion experiments below, WT mice were also treated with or without poly(I:C) 3 h prior to analysis. While poly(I:C) did not alter the expression of the Nrf2-inducible genes in peripheral blood cells, the addition of CDDO-Im significantly increased the expression of *HMOX1* and *NQO1* (Figure 1).

### 3.2. CDDO-Im Inhibits RBC Alloimmunization in a Murine Transfusion Model

**Figure 2:**
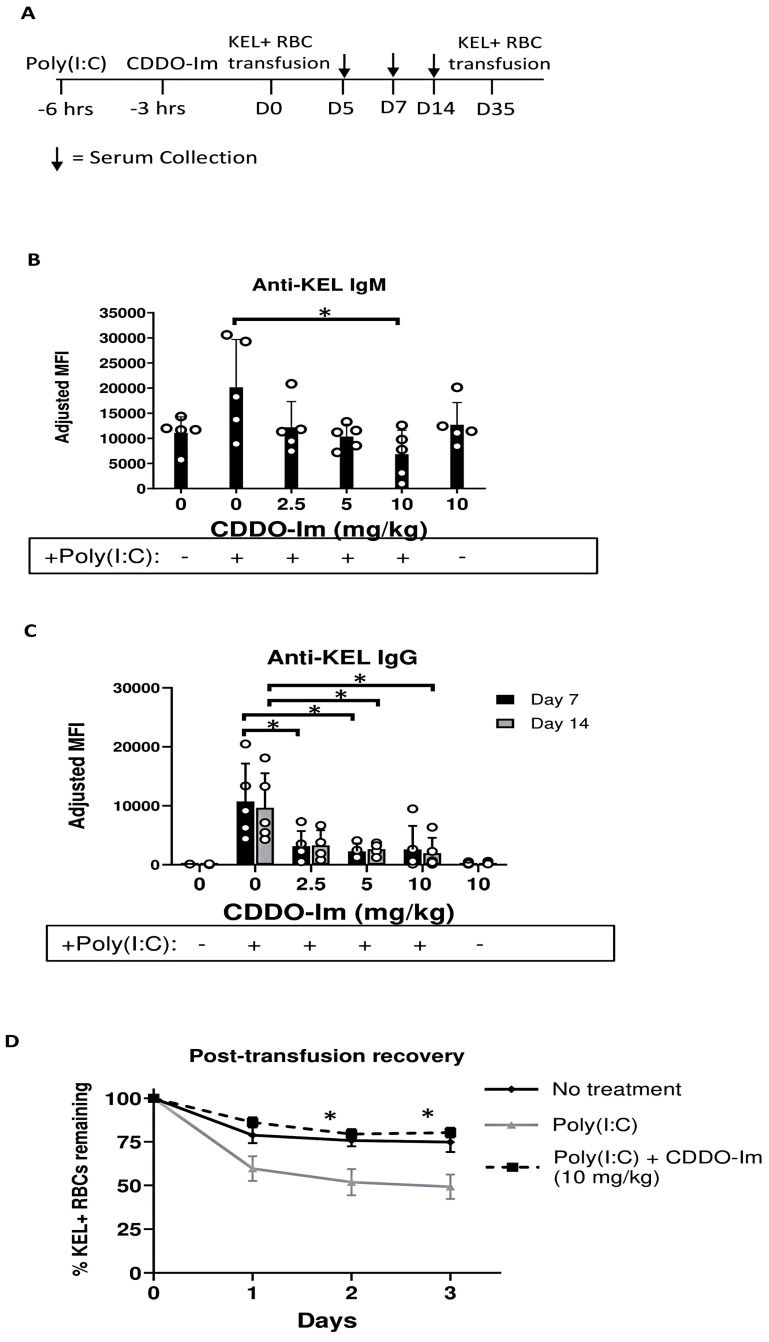
CDDO-Im inhibits inflammation-induced RBC alloimmunization. WT mice were treated with or without poly(I:C) and/or CDDO-Im 3 and 6 h before transfusion, respectively, with KEL + RBCs. (**A**) An experimental timeline showing the schedule of CDDO-Im and poly(I:C) treatments, transfusions, and serum collections. (**B**) Serum anti-KEL IgM levels measured 5 days after transfusion. (**C**) Serum anti-KEL IgG levels measured 7 and 14 days after transfusion. (**D**) The post-transfusion recovery of KEL+ RBCs in peripheral blood 1–3 days after a second transfusion, measured by flow cytometry. The data shown are from one experiment, representative of three independent experiments with five mice per group. * *p* < 0.05 by the Kruskal–Wallis test with Dunn’s post-test for comparison of poly(I:C) vs. poly(I:C) + CDDO-Im groups. (**B**,**C**) * *p* < 0.05 by the Kruskal–Wallis test with Dunn’s post-test.

To investigate the role of Nrf2 activation by CDDO-Im in RBC alloimmunization, we measured anti-RBC antibody responses in a mouse transfusion model. RBCs collected from transgenic mice expressing the human KEL glycoprotein specifically on RBCs (KEL+ RBCs) were leukoreduced and transfused into recipient WT mice treated with or without CDDO-Im and/or poly(I:C) 6 h and 3 h prior to transfusion, respectively (Figure 2A). Compared to mice treated with only poly(I:C), mice treated with poly(I:C) and 10 mg/kg CDDO-Im produced lower levels of anti-KEL IgM 5 days after transfusion (Figure 2B). In addition, poly(I:C) induced anti-KEL IgG antibody production 7 and 14 days after transfusion, while increasing doses of CDDO-Im significantly inhibited poly(I:C)-induced alloimmunization. Treatment with CDDO-Im, without prior poly(I:C) exposure, did not alter antibody responses (Figure 2C).

To determine whether the anti-KEL antibodies were functional, we examined the degree to which anti-KEL antibodies clear transfused RBCs from circulation. Previously transfused mice were re-transfused with fluorescently labeled KEL+ and control WT RBCs 35 days after the initial transfusion. The post-transfusion recovery of transfused RBCs was measured by flow cytometry. While approximately half of the KEL+ RBCs transfused to poly(I:C)-treated mice were cleared from circulation 2–3 days after transfusion, the post-transfusion recovery of KEL+ RBCs in mice treated with poly(I:C) and CDDO-Im was significantly increased, similar to the recovery in WT mice not treated with poly(I:C) (Figure 2D). These results indicate that CDDO-Im inhibits the production of RBC alloantibodies that mediate the clearance of antigen-specific RBCs.

### 3.3. CDDO-Im Inhibition of RBC Alloimmunization Is Nrf2-Dependent

**Figure 3:**
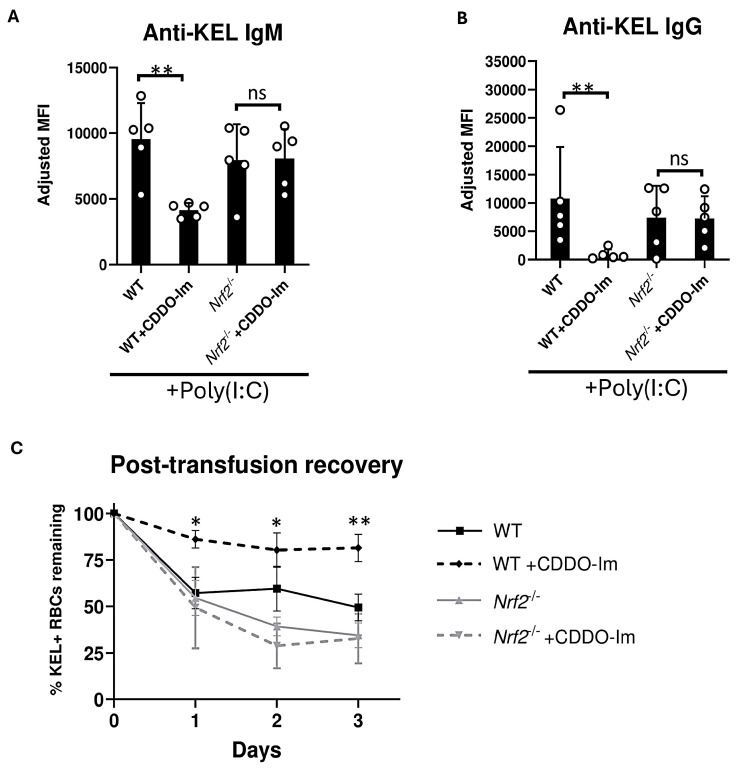
Nrf2 activation inhibits inflammation-induced RBC alloimmunization. WT and *Nrf2^-/-^* mice were treated with poly(I:C), 3 h before transfusion with KEL+ RBCs. Mice were treated with or without CDDO-Im 6 h before transfusion. (**A**) Serum anti-KEL IgM levels measured 5 days after transfusion. (**B**) Serum anti-KEL IgG levels collected 7 and 14 days after transfusion. (**C**) Post-transfusion recovery of KEL+ RBCs in peripheral blood 1–3 days after a second transfusion, measured by flow cytometry. Data are from one experiment, representative of 3 independent experiments with 5 mice per group. (**A**,**B**) ** *p* < 0.01 by Kruskal–Wallis test with Dunn’s post-test. ns, non-significant. (**C**) * *p* < 0.05, ** *p* < 0.01 by Kruskal–Wallis test with Dunn’s post-test for comparison of WT + CDDO-Im vs. *Nrf2^-/-^* + CDDO-Im groups.

Given the possibility that CDDO-Im could alter alloimmunization by Nrf2-independent mechanisms, we examined effects of CDDO-Im on anti-KEL antibody production in Nrf2-deficient (*Nrf2*^-/-^) mice. WT and *Nrf2*^-/-^ were treated with poly(I:C) 3 h prior to transfusion. Treatment with CDDO-Im 6 h prior to transfusion inhibited anti-KEL IgM and IgG production in WT mice. However, CDDO-Im did not alter anti-KEL IgM and IgG production in *Nrf2*^-/-^ mice (Figure 3A,B). When examining post-transfusion recovery, *Nrf2*^-/-^ mice treated with and without CDDO-Im had a reduced recovery of re-transfused KEL+ RBCs compared to CDDO-Im-treated WT mice (Figure 3C). These results indicate that CDDO-Im regulates RBC alloimmunization by activating Nrf2.

### 3.4. CDDO-Im Inhibits Cytokine Production in Mice

**Figure 4:**
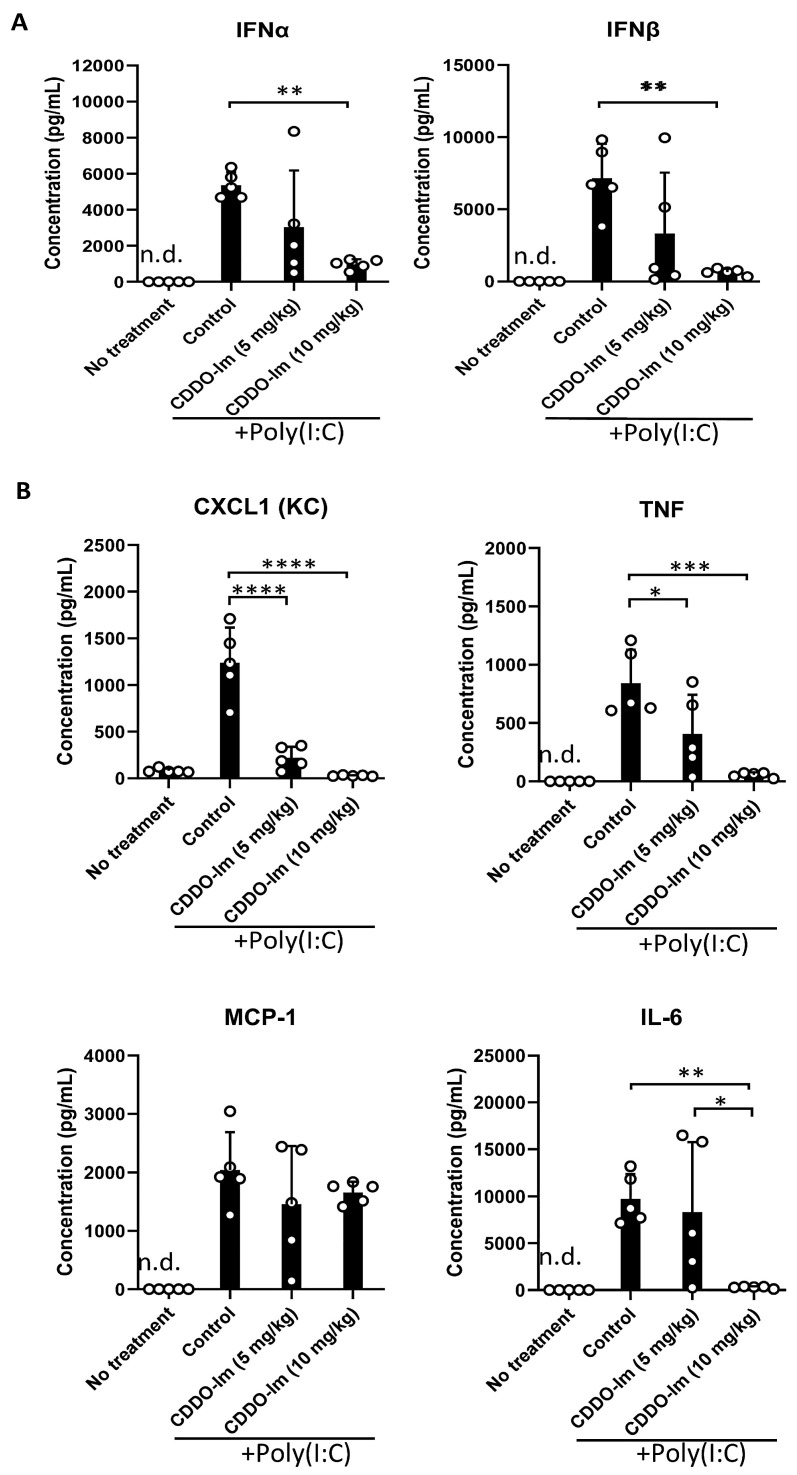
CDDO-Im regulates IFNα/β and NFκB-induced cytokine production in mice. WT mice were treated (i.p.) with 5–10 mg/kg CDDO-Im and/or 100 µg poly(I:C) 6 h and 3 h prior to analysis, respectively. (**A**) IFNα, IFNβ, and (**B**) NF-κB-induced cytokines CXCL1, TNF, MCP-1, and IL-6 levels in serum measured by multiplex bead assay. * *p* < 0.05, ** *p* < 0.01, *** *p* < 0.001, **** *p* < 0.0001 by one-way ANOVA with Tukey’s post-test; 5 mice per group; representative of 3 independent experiments; n.d. = not detectable.

Nrf2 activation can inhibit the production of inflammatory cytokines. Given the role of IFNα/β in promoting alloimmunization to KEL+ RBCs [17,18,19,20], we measured IFNα/β and NF-κB-induced cytokines in the serum of mice treated with CDDO-Im and poly(I:C). CDDO-Im pre-treatment inhibited poly(I:C)-induced IFNα and IFNβ levels (Figure 4A). CDDO-Im also inhibited poly(I:C)-induced NF-κB-mediated CXCL1, TNF, and IL-6 production, while MCP-1 levels were not significantly affected (Figure 4B).

### 3.5. CDDO-Im Promotes Expression of Nrf2-Activated Genes and Inhibits IFNα/β Signaling in Human Macrophages

**Figure 5:**
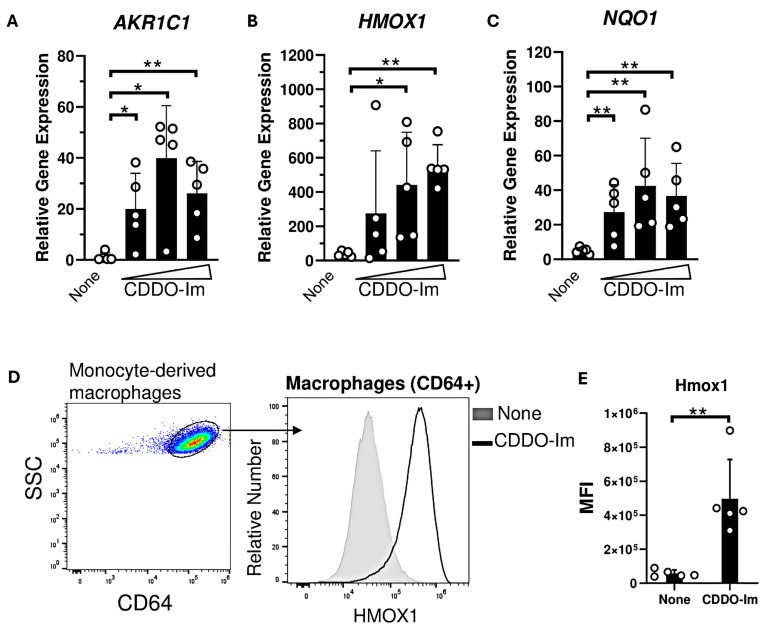
CDDO-Im induces Nrf2-activated gene expression in human macrophages. Human monocyte-derived macrophages were treated with CDDO-Im for 18 h. (**A**) *AKR1C1*, (**B**) *HMOX1*, and (**C**) *NQO1* expression in macrophages treated with either 0, 0.2, 0.4, or 0.8 µM CDDO-Im, measured by RT-qPCR. (**D**) Representative intracellular flow cytometric analysis of Hmox1 expression by CD64+ macrophages treated with or without 0.8 µM CDDO-Im, gated on live cells (**E**) Cumulative data of Hmox1 expression by macrophages from 5 independent experiments; ** *p* < 0.01 by Student’s *t*-test. (**A**–**C**) Each circle represents expression from anindependent experiment, n = 5. * *p* < 0.05, ** *p* < 0.01 by one-way ANOVA with Tukey’s post-test.

Nrf2-activated genes, including *HMOX1* and *NQO1*, are highly expressed in macrophages compared to other innate immune cells [37]. Thus, we examined the degree to which CDDO-Im regulates IFNα/β responses in human monocyte-derived macrophages derived from platelet donors. Macrophages were treated with varying doses of CDDO-Im for 18 h. The expression of Nrf2-activated genes including *AKR1C1*, *HMOX1*, and *NQO1* were significantly elevated compared to untreated macrophages (Figure 5A–C). Intracellular flow cytometry showed that Hmox1 protein expression was elevated in CDDO-Im-treated CD64^+^ macrophages compared to controls (Figure 5D,E).

**Figure 6:**
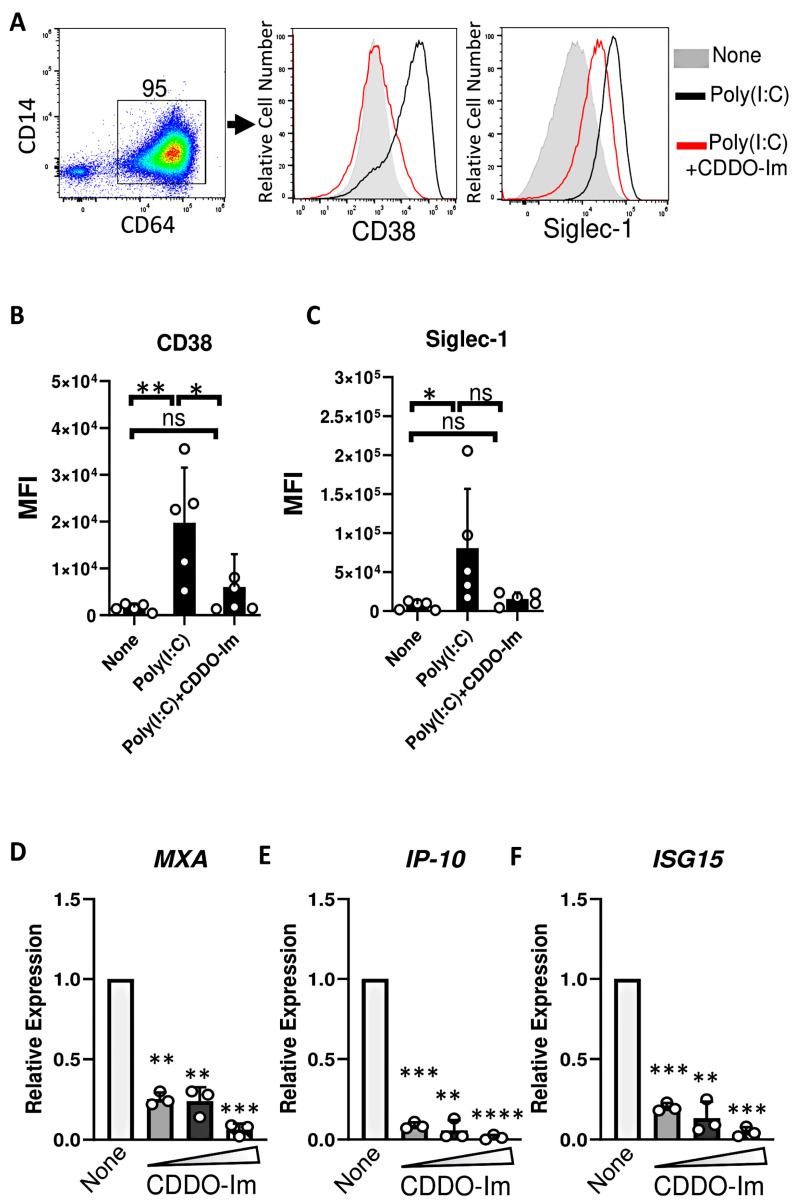
CDDO-Im inhibits IFNα/β activity in human macrophages. Human monocyte-derived macrophages were treated with CDDO-Im for 18 h followed by 1 µg/mL poly(I:C) treatment for 3 (**D**–**F**) or 24 h (**A**–**C**). (**A**) Representative flow cytometric analysis of CD38 and Siglec-1 expression on CD64+ macrophages treated with or without poly(I:C) and 0.8 µM CDDO-Im. (**B**,**C**) Cumulative data of (**B**) CD38 and (**C**) Siglec-1 expression by macrophages from (**A**), n = 5. (**D–F**) Fold expression of ISGs (**D**) *MXA*, (**E**) *IP-10*, and (**F**) *ISG15* in macrophages treated with poly(I:C) and either 0.2, 0.4, or 0.8 µM CDDO-Im, relative to untreated cells (None), measured by RT-qPCR. Each circle represents an independent experiment (n = 3). * *p* < 0.05, ** *p* < 0.01, *** *p* < 0.001, **** *p* < 0.0001 by one-way ANOVA with Tukey’s post-test. ns, not significant.

Given that IFNα/β have been previously shown to induce RBC alloimmunization in murine transfusion models, ISGs were measured in CDDO-Im-treated macrophages. Cells were treated with an IFNα/β stimulus, poly(I:C), which increased the expression of the ISGs, Siglec-1 and CD38, on the cell surface of CD64^+^ macrophages. However, treatment with CDDO-Im prior to poly(I:C) reduced the expression of Siglec-1 and CD38 (Figure 6A–C). In addition, increasing doses of CDDO-Im also inhibited the expression of poly(I:C)-induced ISGs, *MXA*, *IP-10*, and *ISG15*, measured by RT-qPCR (Figure 6D–F).

To determine whether another Nrf2 activator regulates IFNα/β activity in human macrophages, Nrf2-activated genes and ISGs were measured in macrophages treated with sulforaphane. Like CDDO-Im, sulforaphane increased the expression of *HMOX1* and *NQO1*. Prior sulforaphane treatment also inhibited poly(I:C)-induced ISG expression (Supplementary Figure S1).

### 3.6. CDDO-Im Inhibits RBC Alloimmunization in Mice with Pre-Existing Inflammation

**Figure 7:**
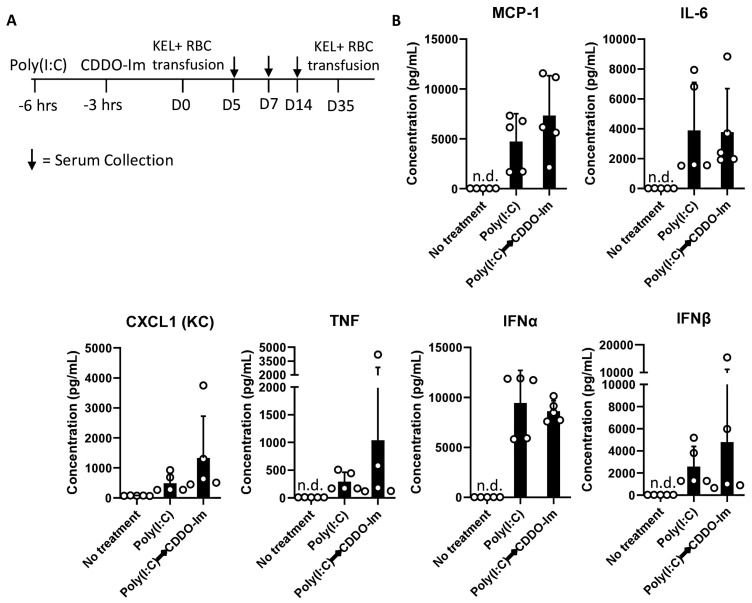

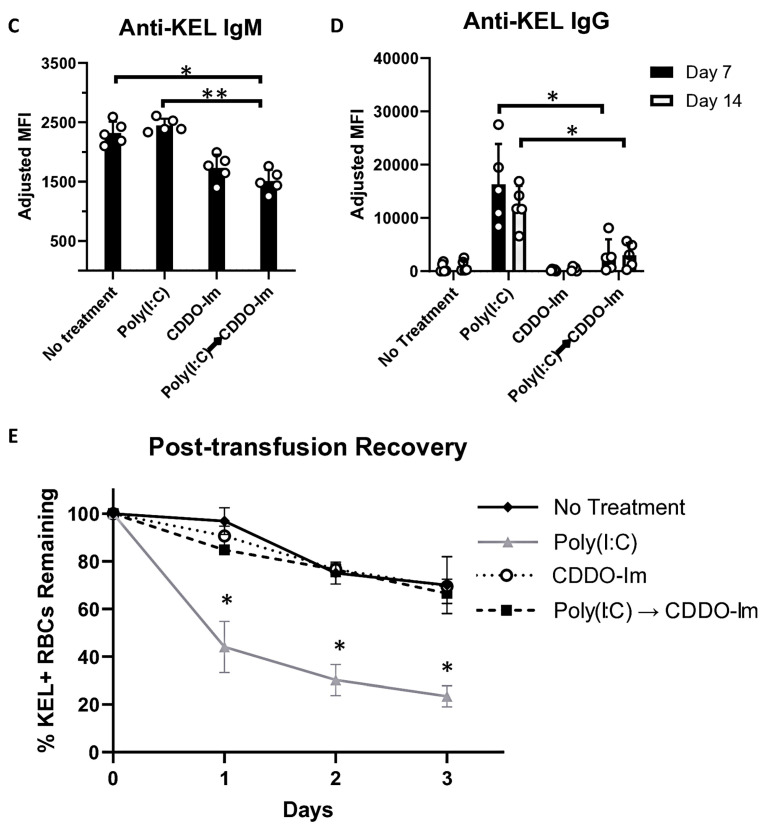
CDDO-Im regulates RBC alloimmunization in mice with pre-existing inflammation. WT mice were treated with or without poly(I:C) and/or CDDO-Im 6 h and 3 h before transfusion, respectively (Poly(I:C) → CDDO-Im), with KEL+ RBCs. (**A**) A timeline of treatments, transfusions, and serum collections. (**B**) Serum cytokines at the time of transfusion. (**C**) Serum anti-KEL IgM levels measured 5 days after transfusion. (**D**) Serum anti-KEL IgG levels measured 7 and 14 days after transfusion. (**E**) The post-transfusion recovery of KEL+ RBCs in peripheral blood 1–3 days after the second transfusion, measured by flow cytometry. The data shown are from one experiment, representative of three independent experiments with five mice per group. (**C**,**D**) * *p* < 0.05, ** *p* < 0.01 by the Kruskal–Wallis test with Dunn’s post-test. (**E**) * *p* < 0.05 by the Kruskal–Wallis test with Dunn’s post-test for a comparison of poly(I:C) vs. Poly(I:C) → CDDO-Im groups.

Given that patients and pre-clinical models with pre-existing inflammation have an increased frequency of alloimmunization [9,10,20,38], we considered whether CDDO-Im also affects alloimmunization in mice with pre-existing IFNα/β and NF-κB activity. Thus, we examined RBC alloimmune responses in WT mice treated with poly(I:C) 3 h before CDDO-Im treatment and 6 h before transfusion with KEL+ RBCs (Figure 7A**)**. At the time of transfusion, IFNα/β and NF-κB-induced cytokine levels were not significantly different between poly(I:C)-treated groups treated with or without CDDO-Im (Figure 7B). Following transfusion, flow cytometric crossmatch analysis indicated that CDDO-Im inhibited anti-KEL IgM and IgG production in WT mice previously treated with poly(I:C) (Figure 7C,D). Re-transfusion with KEL+ and WT RBCs revealed that mice treated only with poly(I:C) prior to the first transfusion had a low post-transfusion recovery of KEL+ RBCs. In contrast, mice treated with CDDO-Im after poly(I:C) treatment had a significantly higher recovery of KEL+ RBCs (Figure 7E). Collectively, the results indicate that CDDO-Im can suppress alloimmunization in mice with pre-existing inflammation.

## 4. Discussion

Currently, the only strategy to mitigate RBC alloimmunization in transfusion recipients is to match multiple RBC antigens expressed by donors and recipients. However, given that there are hundreds of RBC antigens and many variants of Rh group antigens [7], alternate approaches are needed to inhibit alloimmunization and subsequent hemolytic events. While significant progress has been made in identifying inflammatory mechanisms that promote RBC alloimmunization [2], less is known about mechanisms that mitigate it, which is a significant gap in the field. Here, we report that CDDO-Im-mediated Nrf2 activation inhibits RBC alloimmunization in a pre-clinical transfusion model.

Inflammation induced by the viral mimetic poly(I:C) induces and enhances the production of alloantibodies against multiple RBC antigens in pre-clinical studies [15]. We have reported that IFNα/β is required for poly(I:C)-induced alloimmunization [18]. In models of lupus and viral infection, mice lacking IFNα/β production or signaling have profoundly reduced alloantibody responses [17,20]. Thus, given that Nrf2 activation was reported to inhibit IFNα/β responses in models of erythrophagocytosis and bacterial infection [25,39], we tested the degree to which the pharmacologic activation of Nrf2 by CDDO-Im regulates IFNα/β responses to poly(I:C). In human macrophages and mouse blood leukocytes, CDDO-Im treatment prior to poly(I:C) treatment inhibited ISG expression (Figure 6) and IFNα/β production (Figure 4A), respectively. Thus, given the required role of IFNα/β activity in poly(I:C)-induced RBC alloimmunization, CDDO-Im-mediated Nrf2 activation may partly regulate alloimmunization by inhibiting IFNα/β activity. However, the role of other contributing factors, including the CDDO-Im-induced suppression of NF-κB cytokines, cannot be ruled out.

Patients with pre-existing IFNα/β activity, due to autoimmunity, viral infection, or SCD, have an elevated frequency of RBC alloimmunization [9,10,12]. Thus, we tested the degree to which CDDO-Im-mediated Nrf2 activity regulates alloimmunization in mice with pre-existing poly(I:C)-induced inflammation (Figure 7). The result that subsequent CDDO-Im treatment inhibited alloimmunization in the presence of elevated cytokines indicates that CDDO-Im may also prevent cytokine receptor signaling or have cytokine-independent effects. The Nrf2-mediated suppression of IFNα/β-induced Signal Transducer and Activator of Transcription 1 (STAT1) signaling was previously observed in a model of *Klebsiella pneumoniae* infection, where Nrf2 was induced during the erythrophagocytosis of transfused stored RBCs [25]. A further analysis of IFNα/β receptor signaling would determine whether CDDO-Im-mediated Nrf2 activation has a dual role of inhibiting IFNα/β production and signaling.

However, it is notable that Nrf2 activation can also regulate other cytokine responses, including those induced by NF-κB (i.e., TNF, IL-6, and CXCL1) [39]. The transfusion of stored RBCs expressing a chimeric RBC antigen has been shown to promote NF-κB-induced cytokines that may enhance RBC alloantibody responses [40,41]. While this has not been shown for the KEL antigen model reported here, a role for the Nrf2 regulation of non-interferon cytokine responses in RBC alloimmunization cannot be ruled out. In addition, it is notable that CDDO-Im alters Nrf2-independent pathways. For example, CDDO-Im regulates apoptosis [42] and the cell cycle in cancer cells [43], unfolded protein responses [44] and other immune pathways including the mammalian target of rapamycin (mTOR) pathway [45]. Thus, it is possible that CDDO-Im off target effects could influence RBC alloimmunization. However, the inability of CDDO-Im to regulate anti-KEL antibody responses in *Nrf2*-deficient mice (Figure 3) indicates that CDDO-Im inhibits alloimmunization via Nrf2 activation.

Nrf2-induced genes are highly expressed in spleen macrophages [37]. Specifically, red pulp macrophages continually clear senescent circulating RBCs containing oxidized heme, which activates Nrf2 [46]. Thus, we examined CDDO-Im effects on IFNα/β activity in human monocyte-derived macrophages. CDDO-Im induced the expression of multiple Nrf2-stimulated genes (Figure 5) and inhibited poly(I:C)-induced ISG expression (Figure 6), suggesting that immunosuppressive effects of Nrf2 activation can be extended to human macrophages. While macrophages can play a pivotal role in cytokine production and alloimmunization [47], it is possible that CDDO-Im regulates alloimmunization by regulating other immune cells critical for humoral immune responses. Nrf2 activators have been reported to either promote or inhibit T cell and B cell activation, which may depend on the use of specific activators and disease models [48,49]. Heme-induced Nrf2 activation can also inhibit dendritic cell maturation [46]. Subsequent studies utilizing models of *Nrf2* deficiency or activation in specific cell types will clarify the role of cell-specific Nrf2 activity on RBC alloimmunization.

CDDO-Im is one of many described Nrf2-activating compounds. Dimethy fumurate (DMF) is FDA-approved for the treatment of multiple sclerosis [31], and sulforaphane is a Nrf2 activator derived from cruciferous vegetables, including broccoli [30]. While CDDO-Im, DMF, sulforaphane, and many other Nrf2 activators act by altering the principle negative regulator of Nrf2, Keap1, pharmacokinetics, pharmacodynamics, off-target effects, and routes of administration differ [50]. Thus, future studies should delineate the effects of different Nrf2 activators on RBC alloimmunization.

Finally, while prophylaxis for RBC alloimmunization may be beneficial for all transfusion recipients, it is especially impactful for patients with SCD, who have the highest frequency of RBC alloimmunization and a high transfusion burden. Interestingly, Nrf2 activators, including CDDO-Im, sulforaphane, and DMF, have been shown to inhibit vaso occlusion and vascular inflammation in models of SCD [34,35,36]. In addition, a phase 1 trial provided evidence that sulforaphane-containing broccoli sprout homogenates induce the expression of multiple Nrf2 target genes in patients with SCD [51]. Thus, prophylaxis for RBC alloimmunization may be one of many beneficial effects of Nrf2 activators in patients with SCD.

## 5. Conclusions

We report that one Nrf2 activator, CDDO-Im, inhibits RBC alloimmunization in a Nrf2-dependent manner in a pre-clinical transfusion model. To our knowledge, this is the first report of a role for an antioxidant pathway activator in RBC alloimmunization. While CDDO-Im-induced Nrf2 activation may regulate alloimmunization by multiple mechanisms, CDDO-Im inhibits the IFNα/β response, which promotes inflammation-induced RBC alloimmunization. If future studies reveal similar results for other Nrf2 activators in other transfusion models, the findings may lead to human studies examining the prophylactic potential of currently available Nrf2 activators for transfusion recipients.

## Data Availability

Supplementary data may be found in Supplementary Materials available with the online version of this article. For the original data, please contact david.gibb@cshs.org.

## Supplementary Materials

The following supporting information can be downloaded at: https://www.mdpi.com/article/10.3390/antiox14060678/s1, Figure S1: Sulforaphane induces Nrf2-activated gene expression and regulates IFNα/β activity in human macrophages; Table S1: Primer sequences used for RT-q PCR.

## Abbreviations

The following abbreviations are used in this manuscript:

NQO1	NAD(P)H quinone dehydrogenase 1
Hmox1	Heme oxygenase 1
Keap1	Kelch-like ECH-associated protein 1
Nrf2	Nuclear factor erythroid-derived 2-like 2
CDDO-Im	1-[2-cyano-3-,12-dioxooleana-1,9(11)-dien-28-oyl]-imidazole
IFNα/β	Type 1 interferons
ISGs	IFN α/β-stimulated genes
NF-κB	Nuclear factor kappa B
STAT1	Signal Transducer and Activator of Transcription 1
