# Supplementary material for: The Nrf2 Activator CDDO-Imidazole Suppresses Inflammation-Induced Red Blood Cell Alloimmunization
Source: Antioxidants (Basel). 2025 Jun 3;14(6):678. doi: 10.3390/antiox14060678 (PMC12189440; doi:10.3390/antiox14060678)
Supplement: Supplementary file 1 [file antioxidants-14-00678-s001.zip › antioxidants-3637922-supplementary.pdf]

Supplementary Material

The Nrf2 Activator CDDO-Imidazole Suppresses Inflammation-Induced Red Blood Cell Alloimmunization

Che-Yu Chang [1], Rosario Hernández-Armengol [1], Kausik Paul [1], June Young Lee [1], Karina Nance [2], Tomohiro Shibata [3], Peibin Yue [4] and Christian Stehlik [1,2], David R. Gibb [1,5,*]

Affiliations

1. Department of Pathology and Laboratory Medicine, Cedars-Sinai Medical Center, Los Angeles, CA 90048, USA
2. Department of Biomedical Sciences, Cedars-Sinai Medical Center, Los Angeles, CA 90048, USA;
3. Department of Pharmacology, Yokohama City University School of Medicine, Yokohama 236-0004, Japan
4. Department of Medicine, Division of Hematology and Oncology, Cedars-Sinai Medical Center, Los Angeles, CA 90048, USA
5. Division of Transfusion Medicine, Cedars-Sinai Medical Center, Los Angeles, CA 90048, USA

Supplementary Table S1. Primer Sequences used for quantitative PCR.

Primer Name	Forward	Reverse
Mouse *HMOX1*	5'- AGG CTA AGA CCG CCT TCC T -3'	5'- TGT GTT CCT CTG TCA GCA TCA -3'
Mouse *NQO1*	5'- GCC GAA CAC AAG AAG CTG GAA G -3'	5'- GGC AAA TCC TGC TAC GAG CAC T -3'
Mouse GAPDH	5'- CAT CAC TGC CAC CCA GAA GAC TG -3'	5'- ATG CCA GTG AGC TTC CCG TTC AG -3'
Human AKR1C1	5'- CGA GAA GAA CCA TGG GTG GA -3'	5-' GGC CAC AAA GGA CTG GGT CC -3'
Human *HMOX1*	5 -GCT GCT GAC CCA TGA CAC CAA GG-3	5 -AAG GAC CCA TCG GAG AAG CGG AG-3
Human *NQO1*	5'- GAA GAG CAC TGA TCG TAC TGG C-3'	5'- GGA TAC TGA AAG TTC GCA GGG -3'
Human *MXA*	5'- CTC CGA CAC GAG TTC CAC AA -3'	5'- GGC TCT TCC AGT GCC TTG AT -3'
Human *CXCL10* (IP-10)	5-' GGT GAG AAG AGA TGT CTG AAT CC -3'	5-' GTC CAT CCT TGG AAG CAC TGC A -3'
Human *ISG15*	5'- ACT CAT CTT TGC CAG TAC AGG AG -3'	5'- CAG CAT CTT CAC CGT CAG GTC -3'
Human *GAPDH*	5-' TCA CCA GGG CTG CTT TTA AC -3'	5'- ACA AGC TTC CCG TTC TCA G-3'

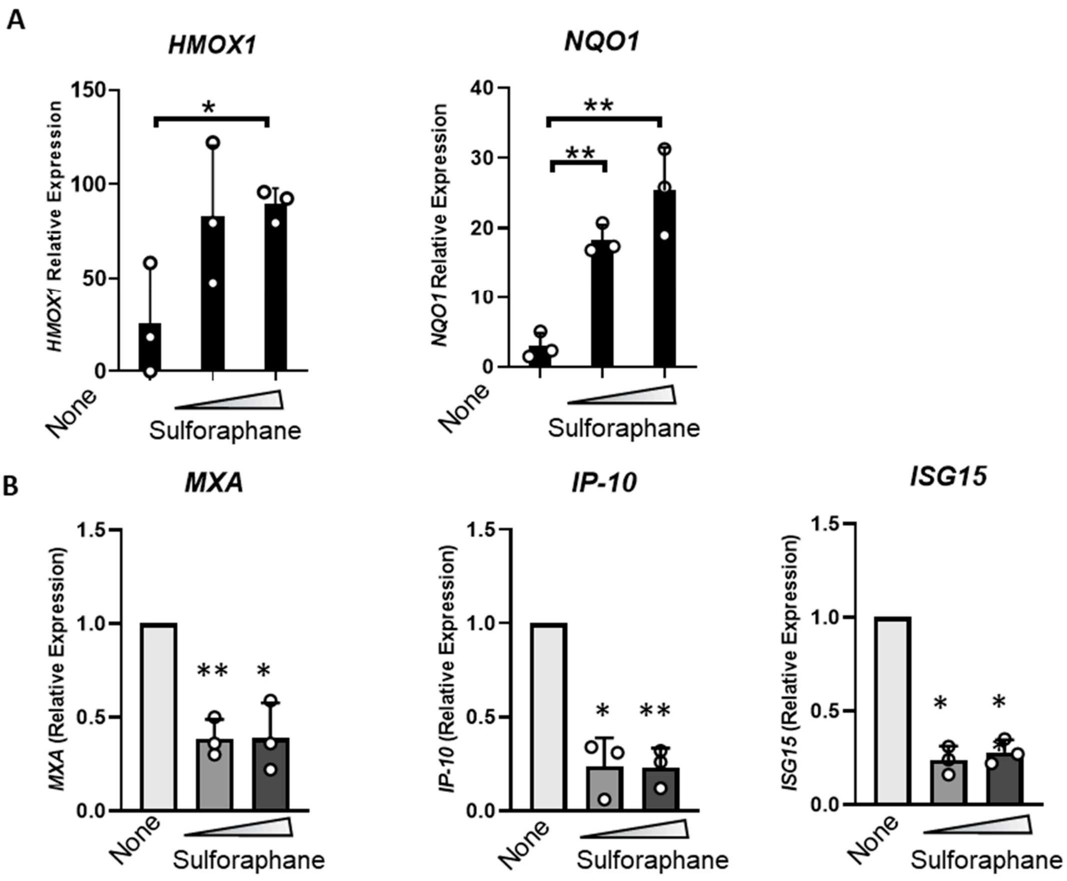

Supplementary Figure S1. Sulforaphane induces Nrf2-activated gene expression and regulates IFNα/β activity in human macrophages. Human monocyte-derived macrophages were treated with 0, 5, or 10 μM sulforaphane for 18 hrs. (**A**) Expression of Nrf2-activated genes, *HMOX1* and *NQO1*, measured by RT-qPCR. (**B**) Following sulforaphane treatment, macrophages were treated with poly(I:C) for 3 hrs. Macrophage fold expression of ISGs, *MXA*, *IP-10*, and *ISG15*, relative to macrophages not treated with sulforaphane, measured by RT-qPCR. *$p<0.05$, **$p<0.01$ by one-way ANOVA with a Tukey's post-test. Each circle represents an independent experiment, n=3.
